# Development of a predictive tool for hemophagocytic lymphohistiocytosis/macrophage activation syndrome risk in adult-onset Still’s disease

**DOI:** 10.3389/fimmu.2026.1709136

**Published:** 2026-07-31

**Authors:** Xinyue Hong, Linzi Meng, Ningqi Dai, Hongxia Shi, Yutong Su, Qiongyi Hu, Yue Sun, Honglei Liu, Jianfen Meng, Jinchao Jia, Mengyan Wang, Chengde Yang, Yaping Wu, Jialin Teng, Huihui Chi

**Affiliations:** 1 Department of Rheumatology and Immunology, Ruijin Hospital, Shanghai Jiao Tong University School of Medicine, Shanghai, China; 2 Department of Rheumatology and Immunology, Traditional Chinese Medicine Hospital of Xinjiang Uygur Autonomous Region, Urumqi, China

**Keywords:** adult-onset Still’s disease, clinical prediction tool, hemophagocytic lymphohistiocytosis, macrophage activation syndrome, predictive model

## Abstract

**Background:**

Hemophagocytic lymphohistiocytosis/macrophage activation syndrome (HLH/MAS) is one of the most common complications of adult-onset Still’s disease (AOSD), and a leading cause of organ failure and mortality in these patients. Early diagnosis and management are crucial but can be challenging. Our study aims to develop a tool for predicting the risk of HLH/MAS in AOSD patients.

**Methods:**

The study recruited AOSD patients admitted to Ruijin Hospital from May 2018 to May 2024. Clinical features and laboratory test results were collected within the first 3 days of hospitalization. Predictor variables were selected using LASSO regression, and a logistic regression model was subsequently developed to predict HLH/MAS. Model performance was assessed through calibration curves, receiver operating characteristic (ROC) curve, decision curve analysis (DCA), and bootstrap internal validation.

**Results:**

This retrospective cohort study included 190 patients with AOSD. Those who developed HLH/MAS had a higher incidence of fever and splenomegaly at admission. They also exhibited more significant reductions in blood cell counts, elevated inflammatory markers, liver dysfunction, and coagulopathy compared to patients without HLH/MAS. We developed the HLH/MAS Risk Score, which included five key predictors—body temperature, platelet count, D-dimer levels, systemic score, and initial glucocorticoid response—to assess the HLH/MAS risk in hospitalized AOSD patients. This model was visualized and demonstrated robust predictive performance, achieving an AUC of 0.86. Comparative analysis with other MAS-related models confirmed that the HLH/MAS Risk Score had competitive discriminative power.

**Conclusions:**

In this study, we developed the HLH/MAS Risk Score, a predictive tool designed to assess the risk of HLH/MAS in AOSD patients during hospitalization. The model demonstrated strong predictive performance and provides a reliable, easy-to-use tool for the early identification of patients at high risk for HLH/MAS. Further external validation is needed to confirm its clinical applicability across diverse populations.

## Introduction

Adult-onset Still’s disease (AOSD) is a rare, polygenic systemic autoinflammatory disease, with hemophagocytic lymphohistiocytosis (HLH) as its most life-threatening complication (1). Previously, macrophage activation syndrome (MAS) was regarded as a secondary form of HLH associated with rheumatic diseases. However, with advancements in research, the distinction between HLH and MAS has become less emphasized, leading us to describe this complication as HLH/MAS. HLH/MAS is caused by severe systemic inflammation, clinically resulting in hyperferritinemia, cytopenia, disseminated intravascular coagulation (DIC), and a high risk of progression to organ failure and mortality (2). HLH/MAS was originally described in pediatrics and has now been recognized in a broader range of primary and secondary forms, including infection, malignancy, and rheumatic diseases such as AOSD and systemic lupus erythematosus (SLE) (3). Early identification and prompt treatment is crucial for improving clinical outcomes. While several retrospective studies have identified potential predictors such as hyperferritinemia, splenomegaly, and cytopenias, no validated screening tool is currently available for predicting HLH/MAS in hospitalized AOSD patients. Existing models lack dynamic information such as response to initial treatment. Our group previously demonstrated that persistent fever after 3 days of high-dose glucocorticoid therapy is an unfavorable prognostic factor in AOSD. Based on this finding, we therefore sought to develop and internally validate the HLH/MAS Risk Score, a model that integrates routine admission data with the initial glucocorticoid response to support early risk stratification.

## Methods

### Reporting

The development and validation of the prediction model adhered to the TRIPOD (Transparent Reporting of a Multivariable Prediction Model for Individual Prognosis or Diagnosis) guidelines to ensure transparency and reproducibility in reporting the findings (4).

### Study design and participants

**Figure 1:**
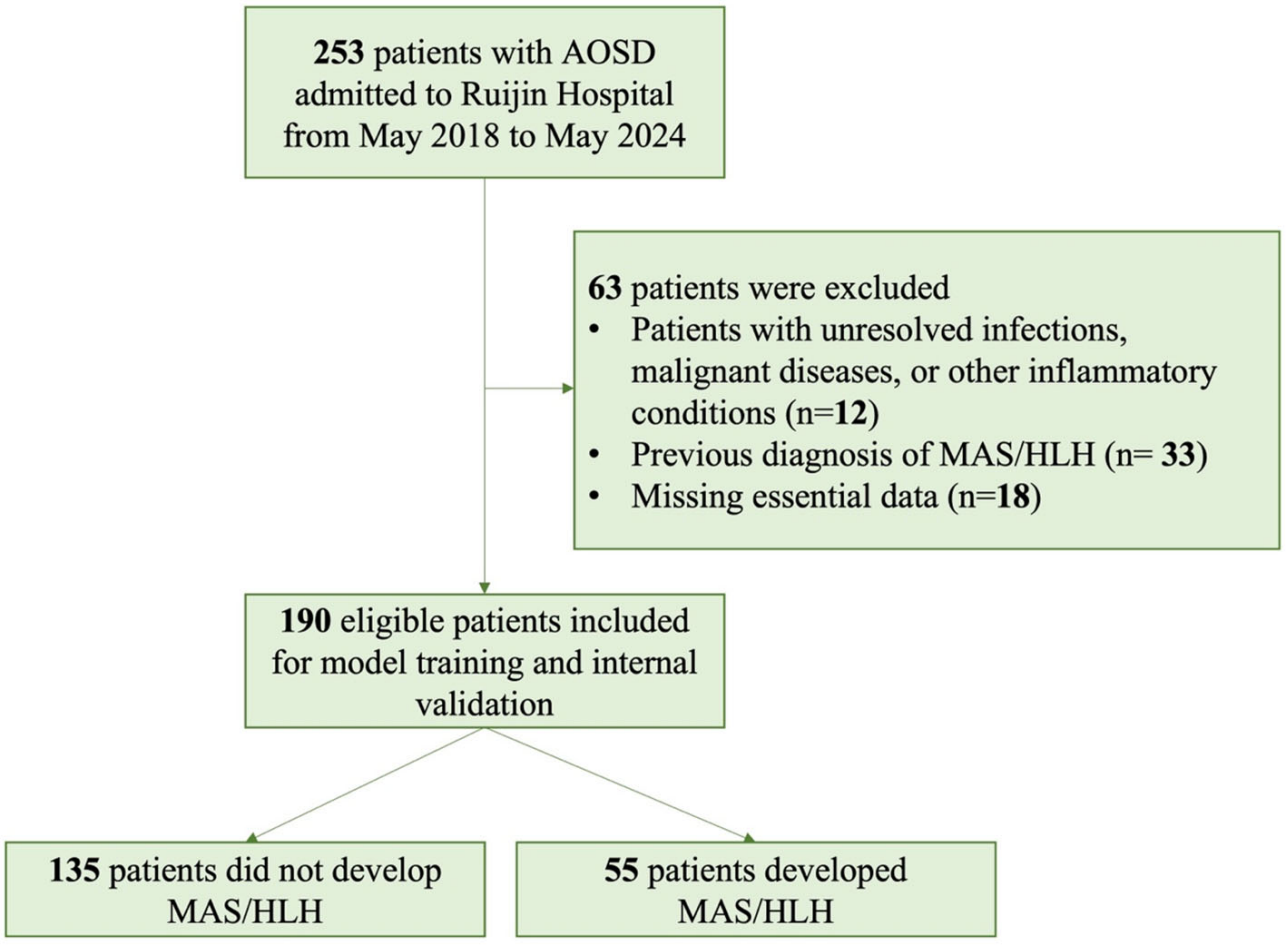
Flowchart of study participants included in the analysis.

This retrospective clinical study was conducted at the Department of Rheumatology and Immunology, Ruijin Hospital, Shanghai Jiao Tong University School of Medicine. We enrolled patients with AOSD who were admitted between May 2018 and May 2024. The diagnosis of AOSD was based on the Yamaguchi criteria (5). Patients with unresolved infections, malignancies, inflammatory diseases, previous diagnosis of HLH/MAS, or missing more than two essential HLH/MAS diagnostic parameters were excluded from the analysis. **Figure 1** outlines the study flow. This study received ethical approval from the Ruijin Hospital Ethics Committee.

### Clinical features and candidate predictor variables

Data collected from patients within the first 3 days of admission included detailed clinical features and key laboratory parameters. The diagnosis of HLH/MAS in this study is based on the HLH-2004 diagnostic criteria, which requires fulfillment of at least five out of the following eight criteria: fever; splenomegaly; cytopenias (affecting more than two of three lineages in the peripheral blood); hypertriglyceridemia and/or hypofibrinogenemia; hemophagocytosis in bone marrow or spleen or lymph nodes without evidence of malignancy; low natural killer (NK) cell activity (according to local laboratory reference); ferritin more than 500 μg/L; sIL-2R more than 2,400 U/mL (6).

AOSD activity was assessed by systemic score, which is based on 12 manifestations, with one point assigned to each manifestation, including fever, typical rash, pleuritis, pneumonia, pericarditis, hepatomegaly or abnormal liver function tests, splenomegaly, lymphadenopathy, leukocytosis (higher than 15,000/mm³), sore throat, myalgia, and abdominal pain (7).

In a previous study, we identified that persistent fever after 3 days of treatment with prednisolone at a dose of 1 mg/kg/day was an unfavorable prognostic factor in AOSD (8). Accordingly, the response to high-dose glucocorticoid was carefully evaluated. A lack of response to high-dose glucocorticoids, assessed after 3 days of treatment, was defined as the persistence of AOSD symptoms—such as fever, new rashes, swollen joints, and pharyngalgia—despite receiving prednisone at a dose exceeding 1 mg/kg/day or its equivalent. These patients often required an escalation in glucocorticoid dosage or the addition of more intensive treatments.

Candidate predictor variables include body temperature, white blood cell count, neutrophil count, lymphocyte count, hemoglobin, platelet count, C-reactive protein (CRP), fibrinogen, D-dimer, activated partial thromboplastin time (APTT), prothrombin time (PT), international normalized ratio (INR), triglycerides, erythrocyte sedimentation rate (ESR), ferritin, soluble interleukin-2 receptor (sIL-2R), alanine aminotransferase (ALT), aspartate aminotransferase (AST), bilirubin, albumin, lactate dehydrogenase (LDH), systemic score, and response to high-dose glucocorticoid therapy. Although hemophagocytosis is recognized as a diagnostic criterion, its detection in tissue biopsies was not included as a variable due to the invasive nature of the procedure, making it unsuitable for dynamic monitoring. Our risk model aims to identify patients at high risk of developing HLH/MAS who may require further investigation, thereby minimizing the need for repeated invasive procedures.

### Model derivation

Variable-specific missing rates were calculated and are presented in **Supplementary Table 3**. All candidate predictors had fewer than 20% missing values, with the majority showing no missing data. The highest missing rate was 15.8% for sIL-2R, while body temperature and CRP each had only two missing values (1.1%). Because the missing data were sporadic and appeared driven by clinician ordering patterns rather than patient clinical status, we treated them as missing at random. The remaining missing data were imputed using a random forest approach to maintain the integrity of the dataset. Variable selection was performed using LASSO regression with α=1 and lambda=1,000 to generate 1,000 distinct values of λ. The coefficient path was plotted to visualize the changes in each feature’s coefficients across the different λ values. To assess the model’s performance, we calculated the area under the receiver operating characteristic curve (AUC) for each λ value. This enabled us to identify the optimal λ and the λ values that fall within one standard error of this optimal point. A sensitivity analysis was performed using complete-case data (patients with no missing values in the five model predictors); the odds ratios were highly consistent with those from the imputed model, indicating that the imputation did not materially influence the results. Linearity of continuous predictors was tested with restricted cubic splines (three knots). Platelet count showed evidence of non-linearity (p = 0.004), whereas the other continuous variables were compatible with a linear assumption (p > 0.3). All continuous predictors were ultimately entered as linear terms to facilitate nomogram construction and bedside calculation. Multicollinearity among the five predictors was assessed with variance inflation factors (VIFs), and all VIFs were well below 2, ruling out significant collinearity.

### Prediction model performance evaluation

A calibration curve was constructed to evaluate the accuracy of the predictive model, while the receiver operating characteristic (ROC) curve was used to assess its diagnostic performance. Decision curve analysis (DCA) was also conducted to determine the model’s clinical utility. Internal validation was performed through bootstrap resampling to assess the model’s stability and generalizability. The optimal probability cutoff for binary classification was identified by maximizing the Youden index on the ROC curve. At this cutoff, sensitivity, specificity, positive predictive value (PPV), and negative predictive value (NPV) were calculated.

### Statistical analysis

Univariate analyses were conducted using independent samples t-tests or Mann–Whitney U tests for continuous variables, depending on data normality. Chi-square tests or Fisher’s exact tests were applied for categorical variables, depending on the expected frequencies. Statistical comparisons were performed using both SPSS (version 26) and R (version 4.4.1), with a significance level of p < 0.05 considered statistically significant.

## Results

### Basic characteristics of AOSD

**Figure 2:**
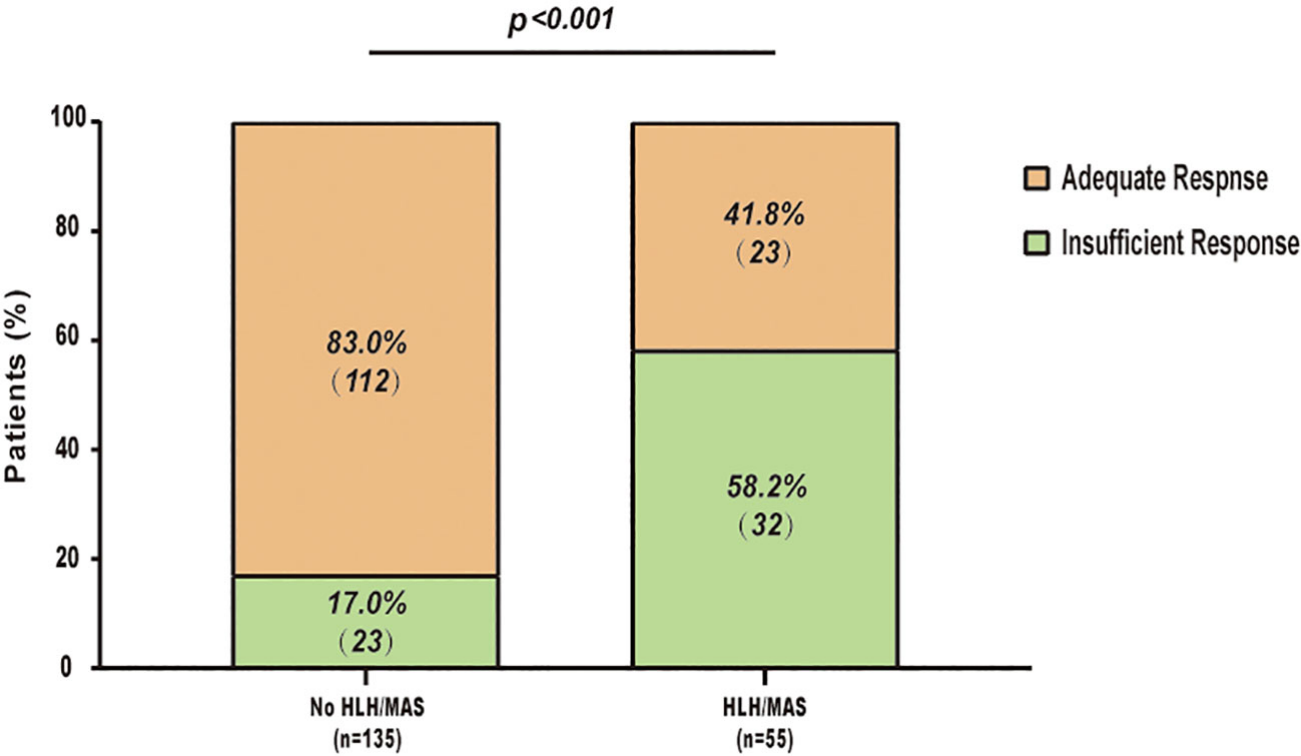
Response to high-dose glucocorticoid therapy within three days of hospital admission.

Consequently, 190 patients were eligible for further analysis. **Table 1** provides detailed baseline and clinical characteristics of the included patients. There were no significant differences in gender and age between two groups. However, the duration of hospitalization was significantly longer for AOSD patients with HLH/MAS (27.0 days) compared to those without (13.0 days). The prevalence of fever at admission was markedly higher in the HLH/MAS group (89.1%) than in the non-HLH/MAS group (56.3%), along with elevated maximum body temperatures. Splenomegaly was more frequent in AOSD patients with HLH/MAS (70.9%) than in those without (33.3%). Additionally, AOSD patients with HLH/MAS had lower lymphocyte counts and hemoglobin levels. The HLH/MAS group also exhibited higher D-dimer levels (3.80 [2.77; 8.48] vs. 1.95 [0.87; 3.39]) and elevated inflammatory markers, including higher ferritin (6577 [1389; 14024] vs. 1989 [566; 4916]) and CRP (77.0 [21.5; 134] vs. 42.0 [17.0; 90.4]), reflecting a more severe inflammatory response at admission. Liver enzymes were higher in the HLH/MAS group, suggesting hepatic impairment. Moreover, the systemic score was significantly higher in the HLH/MAS group, indicating increased disease activity. No significant differences were observed in white blood cell count, neutrophil count, or ESR. Importantly, as shown in **Figure 2**, the initial response to glucocorticoid treatment was significantly higher in patients without HLH/MAS.

**Table 1:** Characteristics of the patients.

Characteristics	Overall (n=190)	AOSD without MAS (n=135)	AOSD with MAS (n=55)	Test statistic*	*p* value
Female	150 (78.9%)	105 (77.8%)	45 (81.8%)	0.384	0.536
Age	38.0 [30.0;51.0]	37.0 [29.0;52.0]	41.0 [30.0;50.0]	−0.876	0.381
Hospitalization duration, day	16.0 [10.0;26.5]	13.0 [9.00;18.5]	27.0 [20.0;43.0]	−6.893	<0.001
Fever	125 (65.8%)	76 (56.3%)	49 (89.1%)	18.674	<0.001
Body temperature maximum, °C	38.5 [36.7;39.2]	38.0 [36.5;39.0]	39.0 [38.3;39.8]	−4.585	<0.001
Splenomegaly	84 (44.2%)	45 (33.3%)	39 (70.9%)	22.371	<0.001
White blood cell count, ×10^9^/L	11.3 [8.29;16.0]	11.3 [8.68;16.5]	11.4 [7.21;15.6]	−1.081	0.280
Neutrophil count, ×10^9^/L	9.03 [6.03;14.0]	8.94 [6.26;14.2]	9.08 [5.97;13.8]	−0.646	0.518
Lymphocyte count, ×10^9^/L	1.11 [0.76;1.81]	1.24 [0.90;1.86]	0.87 [0.60;1.23]	−3.710	<0.001
Hb, g/L	109 [95;124]	112 [98;125]	100 [89;119]	−3.101	0.001
PLT, ×10^9^/L	263 [193;346]	280 [224;354]	178 [119;293]	−4.390	<0.001
CRP, mg/L	52.5 [18.0;116]	42.0 [17.0;90.4]	77.0 [21.5;134]	−2.394	0.017
Fg, g/L	4.40 [2.92;5.60]	4.50 [3.10;5.60]	4.10 [2.75;5.55]	−1.088	0.276
D-dimer, mg/L	2.50 [1.11;4.41]	1.95 [0.87;3.39]	3.80 [2.77;8.48]	−5.025	<0.001
ESR, mm/h	41.0 [20.0;73.5]	41.0 [20.0;70.5]	41.0 [20.5;77.0]	−0.575	0.566
Ferritin, ng/ml	2503 [798;7962]	1989 [566;4916]	6577 [1389;14024]	−3.713	<0.001
ALT, IU/L	31.0 [17.0;80.2]	27.0 [16.0;60.5]	45.0 [22.0;137]	−2.703	0.007
AST, IU/L	42.0 [26.2;79.5]	36.0 [23.0;60.5]	65.0 [41.5;157]	−4.792	<0.001
Systemic score	6.00 [5.00;8.00]	6.00 [4.00;7.00]	8.00 [7.00;9.00]	−5.850	<0.001

Data are presented with n (%) or median [Q1, Q3]. The categorical variables between groups were compared using the Chi-square or Fisher’s exact tests, and the continuous variables were compared using an independent t or Mann–Whitney U tests.

Hb, hemoglobin; PLT, platelet; CRP, C-reactive protein; Fg: fibrinogen; ESR, erythrocyte sedimentation rate; ALT, alanine aminotransferase; AST, aspartate aminotransferase.

*For categorical variables (Female, Fever, Splenomegaly), the test statistic is the Chi-square value (χ²) from Pearson’s Chi-square test. For continuous variables (presented as median [IQR]), the test statistic is the Z value from the Mann–Whitney U test.

### Prediction model for HLH/MAS risk

**Figure 3:**
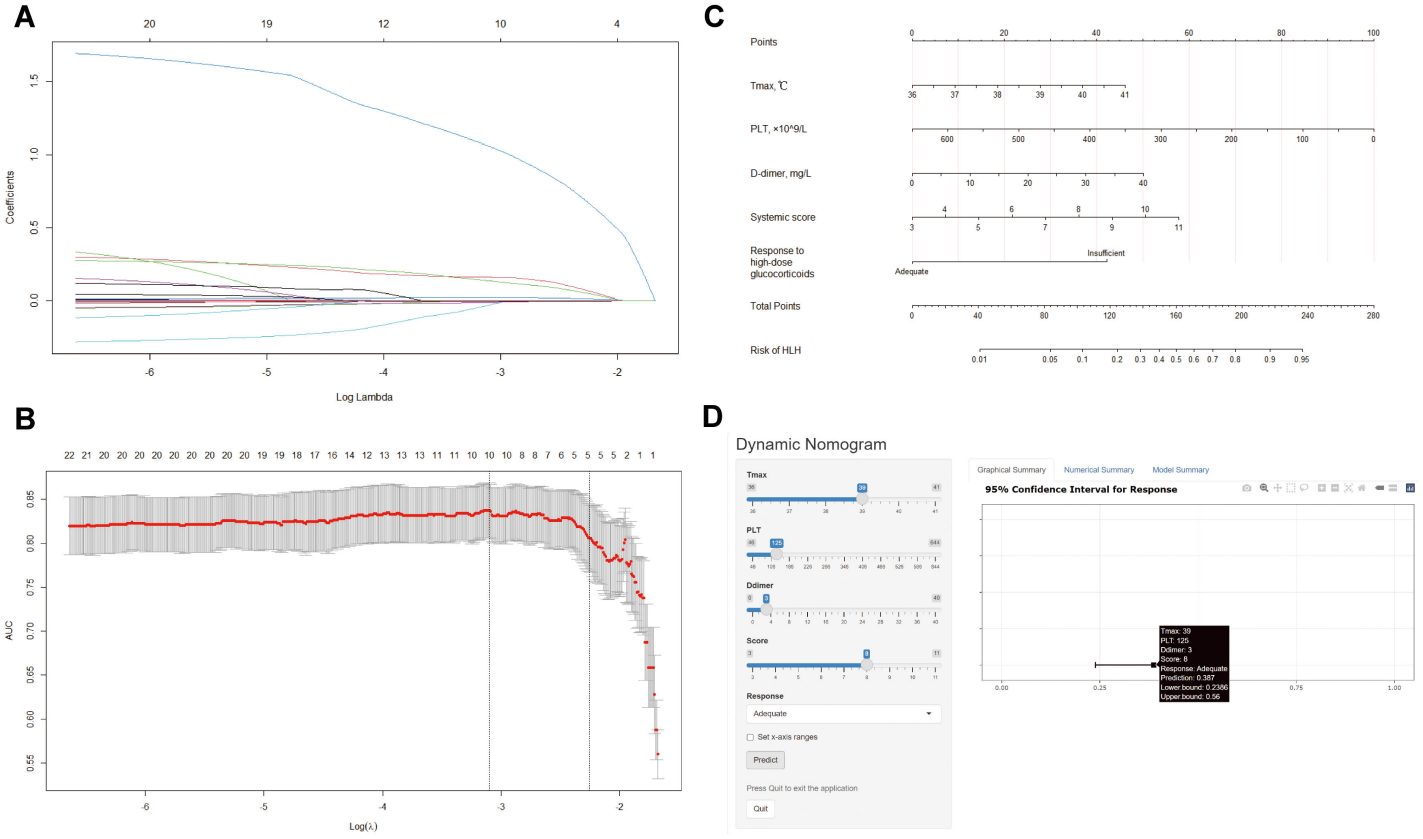
Variable selection and prediction model. **(A)** LASSO path plot. **(B)** Cross-validation curve plot. **(C)** Nomogram for the prediction model. To use the nomogram, locate the patient’s value on each predictor axis and draw a vertical line upward to the “Points” axis to obtain the corresponding score. Sum the five individual scores to get the Total Points, and then locate the Total Points on its axis and draw a vertical line downward to the “Risk” axis to read the predicted probability of HLH/MAS. For example, a patient with a body temperature of 39 °C, platelet count of 125×10^9^/L, D-dimer of 3 mg/L, systemic score of 8, and an adequate glucocorticoid response would have a predicted risk of approximately 38.7% (95% CI: 23.9%-56.0%). At the optimal cutoff, a risk estimate exceeding 0.233 would generally prompt consideration of prompt HLH/MAS evaluation. **(D)** Online dynamic nomogram schematic diagram.

We conducted LASSO regression after the univariate analysis for variable selection, as shown in **Figures 3A, B**. This process resulted in a final model with five predictors: body temperature, platelet count, D-dimer, systemic score, and the initial response to high-dose glucocorticoid treatment upon admission. The optimal λ was 0.10439, with an AUC of 0.8065 and a standard error (SE) of 0.03845. The model included five non-zero variables. It was represented in a nomogram, as shown in **Figure 3C**. A logistic regression model was built with the five selected predictors (**Table 2**). All VIFs were <2, confirming no serious multicollinearity (temperature max: 1.38; platelet: 1.10; D-dimer: 1.19; systemic score: 1.46; glucocorticoid response: 1.14). We have also developed an online dynamic nomogram: https://hxy11235.shinyapps.io/DynNomapp/. We presented a schematic of the online dynamic nomogram in **Figure 3D**. To facilitate the calculation of scores, we have simplified the model in **Supplementary Figure 1**.

**Table 2:** Multivariable logistic regression analysis for the prediction of HLH/MAS in AOSD.

Predictors	β	SE	Wald Z	OR(95% CI)	P
Maximum body temperature, °C	0.3563	0.1569	2.27	1.43 (1.05-1.95)	0.023
Platelet count, ×10^9^/L	−0.0059	0.0019	−3.17	0.994 (0.991-0.998)	0.002
D-dimer, mg/L	0.0484	0.0292	1.66	1.05 (0.99-1.11)	0.097
Systemic score	0.2789	0.1211	2.30	1.32 (1.04-1.68)	0.021
Insufficient glucocorticoid response	1.6300	0.4233	3.85	5.10 (2.23-11.70)	<0.001

SE, standard error; OR, odds ratio; CI, confidence interval. The linear predictor equation is: logit(p) = −15.9902 + 0.3563 * Tmax + −0.0059 * PLT + 0.0484 * Ddimer + 0.2789 * Score + 1.63 * Response.

### Prediction model performance

**Figure 4:**
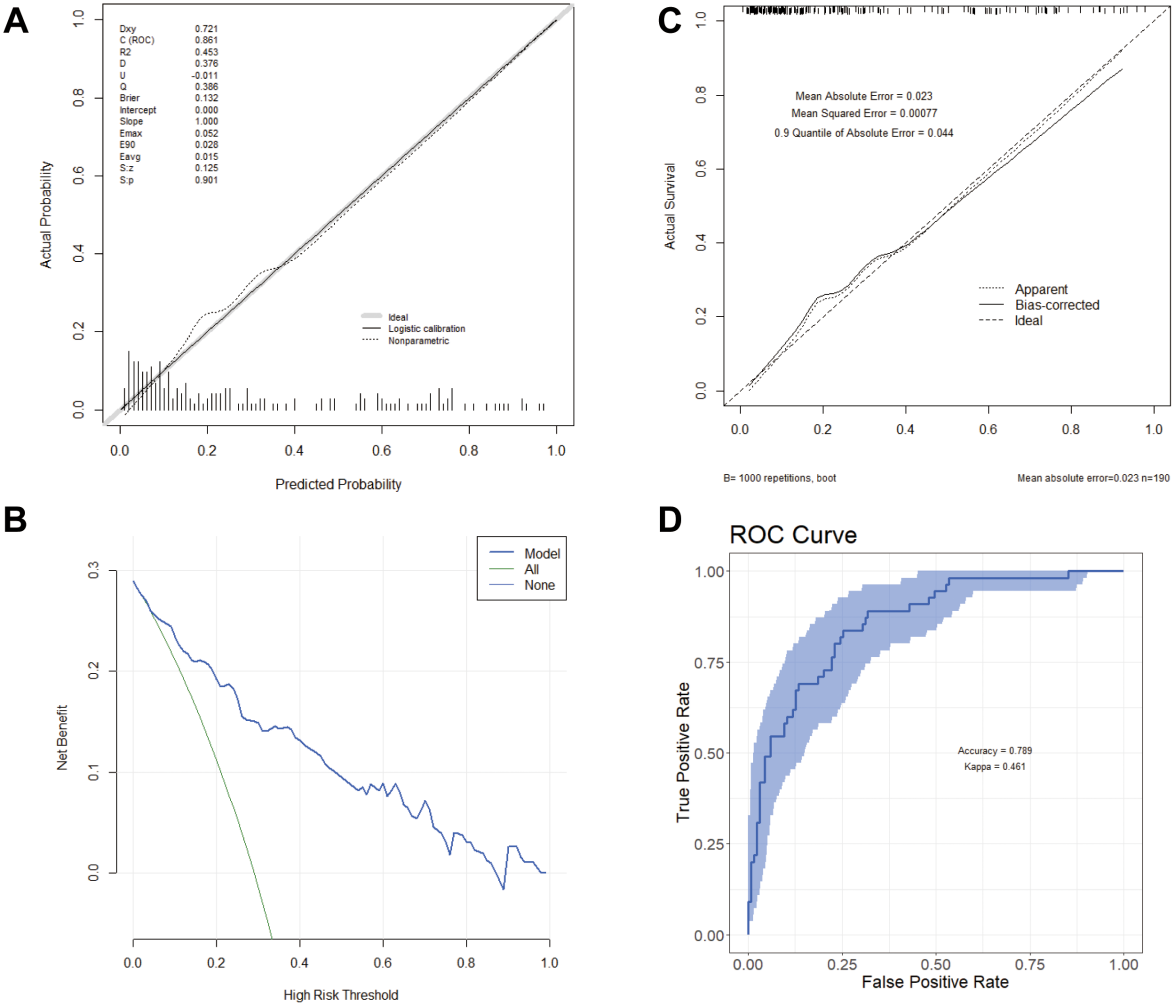
Evaluation and internal validation of the prediction model. **(A)** Calibration curve. **(B)** DCA curve. **(C)** Calibration curve through bootstrap. **(D)** ROC curve through bootstrap.

Calibration curve as shown in **Figure 4A**, the calibration slope is 1, indicating that the model’s predicted probabilities are highly consistent with the observed outcomes. The Brier score of 0.132 reflects good predictive accuracy. Notably, for predicted probabilities greater than 0.4, the calibration curve closely follows the perfect calibration diagonal, suggesting that the model performs well within this range. **Figure 4B** depicts the decision curve analysis, which evaluates the net benefit across various probability thresholds. This analysis provides insights into the model’s clinical utility by showing how its predictions translate into actionable clinical decisions, highlighting its potential effectiveness in practice.

**Figures 4C, D** display the results of the internal validation of the prediction model using bootstrap methods. The calibration curve in **Figure 4C** indicates a well-calibrated model, with a mean absolute error of 0.023 reflecting high accuracy in the predicted probabilities. **Figure 4D** presents the ROC curve with an area under the curve (AUC) of 0.86, demonstrating the model’s strong discriminative ability. Based on the Youden index, we identified an optimal probability cutoff of 0.233, which yielded a sensitivity of 83.6% and a specificity of 74.8%. When patients were stratified by this threshold, HLH/MAS occurred in 11.6% of the low-risk group (<0.233) and 59.4% of the high-risk group (≥0.233), corresponding to a negative predictive value of 88.4% and a positive predictive value of 59.4%, respectively. The model achieved an overall accuracy of 0.789 and a kappa statistic of 0.461, highlighting its effective classification performance and agreement beyond chance.

**Figure 5:**
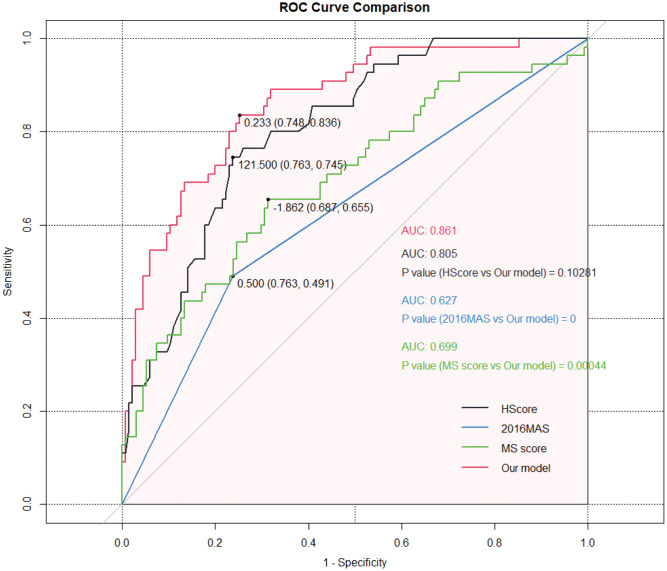
Comparison of ROC curves with other HLH/MAS models.

**Figure 5** compares the ROC curves of HLH/MAS Risk Score with those of other HLH/MAS diagnostic models at admission, including the 2016 MAS criteria, HScore, and MS score models (9–11). These criteria are summarized in **Supplementary Table 1**. This comparison highlights the performance of our model relative to these established models in predictive values. By examining the area under the curve (AUC) for each model, we provide a clear assessment of our model’s discriminative ability about its counterparts. We also conducted a systematic review of published predictors and models for MAS/HLH in AOSD, which is summarized in **Supplementary Table 2**. Notably, none of these studies have developed a validated, easily applicable baseline risk score tailored to hospitalized AOSD patients, further highlighting the novelty of our HLH/MAS risk score.

## Discussion

MAS is the most common and challenging severe complication in AOSD, requiring early recognition and prompt treatment, as emphasized by the recent EULAR/PReS recommendations (12). In this study, we conducted a retrospective consecutive cohort to develop an HLH/MAS risk prediction model for hospitalized AOSD patients, utilizing admission variables. A consecutive cohort design preserves the temporal relationship between predictors and outcomes, avoids selection bias, and maintains the natural incidence of the outcome. This complication can occur at disease onset, during treatment, or even in remission. Our analysis reveals several key differences between AOSD patients with and without MAS at admission. Patients with HLH/MAS exhibited a higher prevalence of fever, more pronounced splenomegaly, and significantly elevated inflammatory markers, including ferritin and CRP. They also had higher D-dimer levels, indicating coagulopathy, along with more severe hepatic impairment. Interestingly, differences in white blood cell count, neutrophil count, and ESR were not statistically significant, possibly due to the variability of these markers at admission, which may have balanced out during statistical analysis. Additionally, our study validated and analyzed the laboratory indicators proposed by the 2022 EULAR/ACR points for the early diagnosis of suspected HLH/MAS (13).

The predictive model for HLH/MAS risk was constructed using LASSO regression, a method that helps to manage the complexity of high-dimensional data. This model identified five significant predictors: body temperature, PLT count, D-dimer levels, systemic score, and initial response to high-dose glucocorticoid treatment. Among these, fever, blood cell counts, coagulation function, and AOSD disease activity are widely recognized common indicators associated with the occurrence of MAS (14). This highlights the strong clinical applicability of our HLH/MAS Risk Score.

The nomogram derived from this logistic regression model, shown in **Figure 3C**, provides a practical tool for clinicians to assess the risk of HLH/MAS. Furthermore, the development of an online dynamic nomogram enhances its clinical utility, enabling real-time and individualized risk evaluation, thereby supporting more informed decision-making in clinical practice. In the future, this scoring system could potentially be integrated as a plugin to combine with patients’ laboratory results, allowing for even more precise and dynamic risk assessment.

To our knowledge, the HLH/MAS Risk Score is the first internally validated prognostic model designed to predict HLH/MAS risk in hospitalized AOSD patients. The 2016 Classification Criteria and the MS score had limited predictive accuracy in our cohort (9, 11); the HLH-2004 criteria require extensive testing for confirmation (6); and the HScore relies on bone marrow analysis, which is rarely available at admission (10). Our review of published predictors (**Supplementary Table 2** (15–31)) identified several candidate biomarkers, including nCD64, sIL-2R, and PTX3, but none have been translated into a validated risk tool. A key feature of our model is the inclusion of the early glucocorticoid response, which captures treatment-refractory inflammation and added independent predictive value (8).

Several limitations of this study should be noted. First, the study was conducted at a single center without external validation, which may limit the generalizability. Furthermore, the study cohort consisted exclusively of Chinese patients, thus potential differences in genetic background or environmental factors could affect the model’s calibration when applied to other ethnic groups. Therefore, external validation in multicentercenter, multiethnic prospective cohorts is essential before this tool can be recommended for widespread global use. In addition, although the linearity test indicated a possible nonlinear relationship for platelet count, we retained the linear specification for model simplicity; future studies could explore whether non-linear terms or spline-based modeling further improve risk estimation. Additionally, the study was conducted in an AOSD cohort, limiting the applicability of the findings to child-onset Still’s disease. Another significant point is the delay in HLH/MAS diagnosis due to the variability of laboratory indicators, which may not always be present simultaneously and can hinder timely diagnosis. Given these factors, while the model aims to identify high-risk patients upon admission, it is important to recognize that it may not capture all cases of HLH/MAS accurately if key diagnostic criteria are delayed. Despite these limitations, the study’s goal to identify high-risk individuals for HLH/MAS at admission is crucial. A model with lower specificity but higher sensitivity may be appropriate to ensure that high-risk patients are identified and managed promptly.

## Conclusion

In this study, we successfully developed the HLH/MAS Risk Score, a predictive tool for assessing the risk of HLH/MAS in AOSD patients during hospitalization. The model demonstrated strong predictive performance, with an AUC of 0.86, and showed competitive discriminative power when compared to other existing models. The inclusion of key clinical and laboratory predictors such as body temperature, platelet count, D-dimer levels, systemic score, and initial glucocorticoid response provides a reliable and easy-to-use tool for early identification of patients at high risk of HLH/MAS. Early detection using this tool can facilitate timely intervention, potentially improving patient outcomes and reducing the risk of severe complications. Further external validation is needed to confirm its clinical applicability in diverse populations.

## Data Availability

The datasets analyzed during the current study are not publicly available due to the protection of patients**’** privacy but are available from the corresponding author on reasonable request. Requests to access these datasets should be directed to Huihui Chi, chh_210@126.com.

## Supplementary material

Supplementary Figure 1 The simplified HLH/MAS risk score. **(A)** Point values for each variable. **(B)** Total points and predicted risk of HLH/MAS. We assigned each interval the nomogram point value corresponding to its midpoint, rounded to the nearest integer. Total points were linked to predicted probability by a cubic polynomial fitted to the nomogram curve: P = −3.18×10^-7^×Points³ + 1.49×10^-4^×Points² − 1.52×10^-^²×Points + 0.421. This conversion facilitates manual calculation while maintaining agreement with the original logistic model.

Supplementary Table 1 Comparison of our model and existing HLH/MAS criteria. HLH, hemophagocytic lymphohistiocytosis; MAS, macrophage activation syndrome; sJIA, systemic juvenile idiopathic arthritis; AOSD, adult-onset Still’s disease.

Supplementary Table 2 Summary of published studies on predictors and prediction models for MAS/HLH in AOSD. NR: Not reported; AOSD: Adult-onset Still’s disease; RHS: Reactive hemophagocytic syndrome; HLH, Hemophagocytic Lymphohistiocytosis; MAS, Macrophage Activation Syndrome; TG, Triglycerides; PPEs, persistent pruritic eruptions; sJIA, Systemic Juvenile Idiopathic Arthritis; NLR, Neutrophil-to-lymphocyte ratio.

Supplementary Table 3 Missing data rates for candidate predictor variables before imputation (N = 190). sIL-2R, soluble interleukin-2 receptor.

Supplementary Table 4 Sensitivity analysis: complete-case vs. multiple imputation model for HLH/MAS risk.
