# Supplementary material for: Development of a predictive tool for hemophagocytic lymphohistiocytosis/macrophage activation syndrome risk in adult-onset Still’s disease
Source: Front Immunol. 2026 Jul 31;17:1709136. doi: 10.3389/fimmu.2026.1709136 (PMC13473414; doi:10.3389/fimmu.2026.1709136)
Supplement: Supplementary file 1 [file DataSheet1.pdf]

**A**

| Tmax, °C | Points |
|---|---|
| <37 | 0 |
| 37-37.9 | 10 |
| 38-38.9 | 20 |
| 39-39.9 | 30 |
| ≥40 | 40 |

| PLT, ×10^9/L | Points |
|---|---|
| <50 | 100 |
| 50-99 | 90 |
| 100-149 | 80 |
| 150-199 | 70 |
| 200-249 | 70 |
| 250-299 | 60 |
| ≥300 | 50 |

| Response to high-dose glucocorticoids | Points |
|---|---|
| Adequate | 0 |
| Insufficient | 40 |

| D-dimer, mg/L | Points |
|---|---|
| <5 | 0 |
| 5-9.9 | 10 |
| 10-14.9 | 15 |
| 15-19.9 | 20 |
| 20-24.9 | 30 |
| 25-29.9 | 35 |
| 30-34.5 | 40 |
| 35-39.5 | 45 |
| ≥40 | 50 |

| Systemic score | Points |
|---|---|
| 0-3 | 0 |
| 4 | 5 |
| 5 | 15 |
| 6 | 20 |
| 7 | 25 |
| 8 | 35 |
| 9 | 40 |
| 10 | 50 |
| 11 | 60 |

**B**

| Summation of Points: | |
|---|---|
| Tmax | +_______ |
| PLT | +_______ |
| D-dimer | +_______ |
| Systemic score | +_______ |
| Response to high-dose glucocorticoids | +_______ |
| **Total Risk Score =** | |

| HLH/MAS Risk Score | Predicted Risk of HLH/MAS During Hospitalization, % |
|---|---|
| 0-40 | <1 |
| 41-84 | 1-<5 |
| 85-104 | 5-<10 |
| 105-124 | 10-<20 |
| 125-136 | 20-<30 |
| 137-148 | 30-<40 |
| 149-160 | 40-<50 |
| 161-168 | 50-<60 |
| 169-180 | 60-<70 |
| 181-196 | 70-<80 |
| 197-216 | 80-<90 |
| 217-236 | 90-<95 |
| ≥237 | ≥95 |
