# Supplementary material for: Development of a predictive tool for hemophagocytic lymphohistiocytosis/macrophage activation syndrome risk in adult-onset Still’s disease
Source: Front Immunol. 2026 Jul 31;17:1709136. doi: 10.3389/fimmu.2026.1709136 (PMC13473414; doi:10.3389/fimmu.2026.1709136)
Supplement: Supplementary file 2 [file Table1.docx]

**Supplementary table 1**. Comparison of our model and existing HLH/MAS criteria

| Model / Criteria | Indicator | Purpose | Population | Reference |
| --- | --- | --- | --- | --- |
| 2016MAS | Ferritin, platelet count, aspartate aminotransferase, triglycerides, and fibrinogen | Classify criteria for MAS complicating sJIA | Children with suspected MAS, including sJIA and systemic infections | (10) |
| HScore | Known underlying immunosuppression, high temperature, organomegaly, triglyceride, ferritin, serum glutamic oxaloacetic transaminase, and fibrinogen levels, cytopenia, and hemophagocytosis features on bone marrow | Estimate an individual’s risk of having reactive HLH | Patients with bone marrow for suspected HLH | (11) |
| MS score | Central nervous system involvement, haemorrhagic manifestations, active arthritis, platelet count, lactic dehydrogenase, fibrinogen, and ferritin | Identify MAS in patients with sJIA | sJIA patients | (12) |
| HLH/MAS Risk Score | Body temperature, platelet count, D-dimer levels, systemic score, and glucocorticoid response | Predict the risk of HLH/MAS in AOSD patients | Hospitalization AOSD patients | This study |

Abbreviations: HLH: haemophagocytic lymphohistiocytosis; MAS: macrophage activation syndrome; sJIA: systemic juvenile idiopathic arthritis; AOSD: adult-onset Still’s disease.
