# Supplementary material for: Development of a predictive tool for hemophagocytic lymphohistiocytosis/macrophage activation syndrome risk in adult-onset Still’s disease
Source: Front Immunol. 2026 Jul 31;17:1709136. doi: 10.3389/fimmu.2026.1709136 (PMC13473414; doi:10.3389/fimmu.2026.1709136)
Supplement: Supplementary file 3 [file Table2.docx]

**Supplementary table 2**. Summary of published studies on predictors and prediction models for MAS/HLH in AOSD

| Author (Year) | Study design | Population | Sample size (Total;HLH/MAS cases) | Criteria for HLH/MAS | Model type/Statistical approach | Key predictors | Statistical results | Key findings |
| --- | --- | --- | --- | --- | --- | --- | --- | --- |
| Bae et al. (2015)(15) | Retrospective cohort study | AOSD patients | 109;21 | Definite RHS: bone marrow, liver, or lymph node biopsy  Probable RHS: high fever with unexplained progressive cytopenia affecting at least 2 cell lineages, occurrence of RHS during the course of AOSD in spite of proper treatment, and exclusion of malignancy-associated RHS. | Multivariate logistic regression | Low platelet count (<121,000/mm³); Anemia (Hb <10.0 g/dL); Hepatomegaly | Low platelets: OR=1.062 (95% CI 1.02–1.10), AUC=0.98. Anemia: OR=3.378 (95% CI 1.16–9.80). Hepatomegaly: OR=1000 (95% CI 2.11–∞). Presence of ≥2 of these 3 factors yielded AUC=0.87. | Low platelet count, anemia, and hepatomegaly independently predicted RHS in AOSD. AOSD patients with RHS had higher relapse rates but similar long-term survival compared to those without RHS. HLH patients had worse prognosis than RHS patients. |
| Ruscitti et al. (2018)(16) | Retrospective multicenter cohort study | AOSD patients | 119;17 | HLH-2004 / HScore / Batu criteria | Multivariate logistic regression | Lymphadenopathy; Abdominal pain | Lymphadenopathy: OR=7.22 (95% CI 1.49–34.97, p=0.014). Abdominal pain: OR=4.36 (95% CI 1.24–15.39, p=0.022). Systemic score ≥6.5 predicted MAS at admission (sens 75%, spec 68%); ferritin ≥1320.5 ng/mL (sens 91%, spec 60%). | MAS significantly reduced survival in AOSD (p<0.0001). Lymphadenopathy and abdominal pain at diagnosis independently predicted MAS. Higher systemic score and ferritin were associated with MAS occurrence. |
| Yang et al. (2019)(17) | Retrospective cohort study | Hospitalized patients with AOSD | 182;11 | HLH-2004 / HScore | Multivariate logistic regression | Splenomegaly; Pericarditis; Ferritin >2000 ug/l | Splenomegaly: OR=5.748 (95% CI 1.378–23.984, p=0.016). Pericarditis: OR=6.492 (95% CI 1.43–29.461, p=0.015). Ferritin >2000 μg/L: OR=4.715 (95% CI 1.12–19.86, p=0.035). | MAS occurrence significantly reduced survival in AOSD (p=0.018). Splenomegaly, pericarditis, and extremely elevated ferritin at diagnosis independently predicted MAS development. |
| Di Benedetto et al. (2020)(18) | Retrospective analysis of a prospective multicenter cohort | AOSD patients from GIRRCS cohort | 147;26 | HLH-1991/HLH-2004 / HScore / Batu criteria | Multivariate logistic regression; ROC analysis | Ferritin (continuous); Ferritin ≥1225 ng/mL | Ferritin (continuous): OR=1.734 (95% CI 1.094–2.749, p=0.019). Ferritin ≥1225 ng/mL: AUC=0.739 (95% CI 0.652–0.825), sensitivity 88%, specificity 57%. | Ferritin at diagnosis independently predicted MAS. A cutoff of 1225 ng/mL had high sensitivity (88%) for identifying patients who developed MAS. Ferritin also correlated with systemic score and specific clinical features. CRP at the time of diagnosis was predictive of mortality during AOSD. |
| Wang et al. (2020)(19) | Multicenter retrospective cohort study | Hospitalized AOSD patients | 447;55 | HLH-2004/HLH-2009 | Multivariate logistic regression | PLT <100×10⁹/L; AST >120 U/L; TG >3 mmol/L; Ferritin >1500 ng/mL; Hemophagocytosis in bone puncture | PLT <100×10⁹/L: OR=9.546 (p=0.005). AST >120: OR=25.853 (p<0.0001). TG >3: OR=12.983 (p=0.011). Ferritin >1500: OR=5.513 (p=0.050). Hemophagocytosis: OR=18.132 (p=0.001). | MAS was the major cause of death in AOSD (OR=11.71, p<0.0001). Liver dysfunction had extremely high OR (75.744) in univariate analysis. PLT <100×10⁹/L, AST >120 U/L, TG >3 mmol/L, ferritin >1500 ng/mL, and hemophagocytosis in bone puncture were highly associated with MAS occurrence. |
| Gao et al. (2021)(20) | Retrospective cohort study | Hospitalized patients with AOSD | 111;9 | HLH-2004 and HScore | 1) Multivariate logistic regression; 2) Multivariate Cox proportional hazards model | For MAS: Jaundice.  For death: MAS; WBC >20×10⁹/L. | For MAS: Jaundice OR=16.50 (95% CI 2.73–99.82, p=0.002). For death: MAS HR=11.22 (95% CI 3.46–36.38, p<0.001); WBC >20×10⁹/L HR=5.80 (95% CI 1.09–30.92, p=0.040). | Jaundice was an independent predictor of MAS. In AOSD-MAS patients, elevated transaminases, TG, and ferritin often preceded hematologic changes, aiding early recognition. |
| Javaux et al. (2021)(21) | Retrospective multicenter cohort study | Patients with Still's disease | 206;20 | Final clinical judgment of the physician | ROC analysis; Kaplan-Meier survival analysis | For MAS detection: Ferritin; LDH; Fibrinogen; HScore.  For MAS prediction: Baseline ferritin >10,448 μg/L. | LDH >459 U/L: Sens 89.5%, Spec 72.3%, NPV 97.5%. Fibrinogen <4.39 g/L: NPV 94.0%. HScore >135: Sens 90.0%, Spec 85.0%, NPV 98.8%. Ferritin >10,448 μg/L at diagnosis: significantly higher risk of developing MAS (p<0.001). | Extreme hyperferritinemia at SD onset predicts subsequent MAS. Optimized thresholds for ferritin, LDH, fibrinogen, and HScore improve MAS detection. Neurological symptoms and hepatomegaly are clinical clues for MAS. |
| Wan et al. (2021)(22) | Retrospective cohort | AOSD patients who underwent pre‑treatment 18F-FDG PET/CT | 57;8 | HLH-2004 | Logistic regression | MLV_total of LNs; IL‑2R; SUV_max of spleen > 3.55; | MLV_total of LNs > 62.2: AUC=0.855, sens 80.0%, spec 93.9%, OR=27.375 (95% CI 1.117–123,080.200). IL‑2R > 2824 pg/mL: AUC=0.899, sens 75.0%, spec 97.3%. SUV_max of spleen > 3.55: AUC 0.765, sens 100%, spec 53.1%. | MLV_total of LNs > 62.2 on PET/CT was an independent predictor of MAS. Spleen SUV_max correlated with systemic score and may reflect disease severity. Combining PET parameters with IL‑2R could enhance MAS prediction. |
| Ding et al. (2021)(23) | Retrospective cohort study | Untreated AOSD patients with skin involvement | 150;17 | HLH-2009 | Univariate and multivariate Cox proportional hazard regression；Kaplan-Meier | PPEs; AST >120 U/L; Splenomegaly | PPEs: HR=5.519 (95% CI 1.138–26.767, p=0.034). AST >120 U/L: HR=8.084 (95% CI 1.728–37.826, p=0.008). Splenomegaly: HR=21.152 (95% CI 2.263–197.711, p=0.007). | AOSD patients with PPEs had higher MAS incidence than those with evanescent rash (17.4% vs 3.1%). PPEs, AST >120 U/L, and splenomegaly were independent risk factors for MAS; presence of all three factors conferred the highest risk. |
| Ruscitti et al. (2022)(24) | Multicenter retrospective cohort study | Patients with AOSD | 185;22 | HScore / Batu criteria | Univariate and multivariate Cox regression | Smoking habit (current or former) | MAS: Univariate HR=5.98 (95% CI 2.45–14.57, p<0.0001); Multivariate HR=6.21 (95% CI 2.46–15.70, p<0.0001). Parenchymal lung disease: Multivariate HR=3.90 (95% CI 1.36–11.23, p=0.012). Mortality: Univariate HR=4.25 (95% CI 1.33–13.55, p=0.015). | Smoking habit independently predicted MAS, parenchymal lung disease, and mortality in AOSD. Smokers had higher systemic scores and more frequent serositis. Smoking is a negative prognostic factor in AOSD. |
| Antón et al. (2024)(25) | Multicenter retrospective cohort study | Patients with sJIA or AOSD | 326 (sJIA: 219, AOSD: 107);61 | 2016 JIA-MAS criteria / Confirmed pathology | Multivariate logistic regression | sJIA diagnosis (compared to AOSD); Serositis; Need for biologic therapy | sJIA: OR=5.43 (95% CI 1.43–20.6, p=0.013). Serositis: OR=4.55 (95% CI 1.88–11.01, p=0.001). Need for biologics: OR=4.37 (95% CI 1.39–13.7, p=0.011). | MAS was significantly more frequent in sJIA (24.4%) than AOSD (9.5%). sJIA diagnosis, serositis at onset, and requirement for biologic therapy independently predicted MAS development. The three-factor model had moderate discriminative ability AUC=0.767.. |
| Ruscitti et al. (2024)(26) | Multicenter observational prospective cohort study | Patients with Still's disease from GIRRCS and AIDA registries | 597;78 | HLH-1991/ HLH-2004 / HScore / Batu criteria / 2016 JIA-MAS criteria | Multivariate logistic regression | Systemic score(Pouchot score); Liver involvement; Lung disease | Systemic score (continuous): OR=1.24 (95% CI 1.07–1.42, p=0.004). Systemic score ≥7: OR=3.36 (95% CI 1.81–6.25, p<0.001). Liver involvement: OR=1.68 (95% CI 1.48–2.67, p=0.031). Lung disease: OR=2.12 (95% CI 1.14–4.49, p=0.042). | Higher systemic score at diagnosis predicts life-threatening evolution. A score ≥7 indicates increased risk. Among score components, liver involvement and lung disease independently predict poor outcome; lung disease also associated with higher mortality. |
| Zhu et al.(2024)(27) | Retrospective cohort study | Patients with AOSD | 152;70 | 2016 JIA-MAS criteria | Multivariate logistic regression; Nomogram; Compared linear, logistic, lasso, ridge, elastic net models | PTX3; Ferritin; sIL-2R | PTX3 AUC 0.841(0.765,0.917). The combined model (PTX3 + ferritin + sIL-2R) yielded improved predictive accuracy (exact metrics NR). | PTX3 is a novel biomarker for AOSD disease activity and strongly associated with MAS. A combined model of PTX3, ferritin, and sIL-2R significantly improves prediction of MAS over individual markers. PTX3 may also contribute to pathogenesis by inducing NET formation via MAPK pathway. |
| Cheng et al. (2025)(28) | Retrospective case‑control study | AOSD patients | 105;26 | HLH-2004 | Multivariable logistic regression; ROC analysis | NLR  ; AOSD systemic score | NLR (cutoff 17.455): AUC=0.682, sens 38.5%, spec 91.0%. Systemic score (cutoff 5.500): AUC=0.709, sens 84.6%, spec 47.4%. | NLR and systemic score were independent predictors of MAS in AOSD. NLR > 17.455 had high specificity (91.0%); systemic score > 5.5 had high sensitivity (84.6%). Monitoring these parameters may facilitate early recognition of MAS. |
| Wu et al. (2025)(29) | Retrospective cohort | AOSD Patients | Training: 83;48  Validation: 35;NR | HLH-2004 | Logistic regression; ROC analysis; Kaplan-Meier | nCD64 index | Training cohort: AUC=0.877 (cutoff 32.09, sens 0.792, spec 0.971).  Validation cohort: sens 0.786, spec 0.905, PPV 0.846, NPV 0.864.  Combined models: nCD64+ferritin AUC=0.942; nCD64+sIL-2R AUC=0.944. | nCD64 index independently predicts MAS in AOSD. Elevated nCD64 (>32.09) associated with significantly higher 28-day MAS cumulative incidence. Combining nCD64 with ferritin and sIL-2R improves predictive accuracy. |
| Bektas et al. (2026)(30) | Retrospective multicenter cohort study | Patients with Still's disease (without infection) | 110;16 | HLH-2004 | Univariate and multivariate logistic regression | PCT | PCT ≥0.5: Univariate OR=6.4 (p=0.002); Multivariate OR=4.3 (p=0.038). PCT_max ≥0.5: Univariate OR=7.6 (p=0.003); Multivariate OR=5.4 (p=0.020). | Elevated PCT in Still's disease, even without infection independently predicts MAS. Multivariate OR=4.3 (p=0.038) for PCT≥0.5ng/ml and OR=5.4 (p=0.020) for peak PCT ≥0.5ng/ml. |
| Casafont-Solé et al. (2026)(31) | Retrospective cohort study | AOSD patients from two Spanish university hospitals | 64;NR | NR | Descriptive and comparative analysis (no multivariable predictive model for MAS) | Systemic Score ≥7 | SS≥7 (sensitivity: 41.7%, specificity: 90%) | Systemic Score ≥7 was significantly associated with MAS, pericarditis, pleuritis, and renal involvement. It also identified patients more likely to require biologic therapy. The score may aid early risk stratification. |

Abbreviations: NR: Not reported; AOSD: Adult-onset Still’s disease; RHS: Reactive hemophagocytic syndrome; HLH: Haemophagocytic Lymphohistiocytosis; MAS: Macrophage Activation Syndrome; TG: Triglycerides; PPEs: persistent pruritic eruptions; sJIA: Systemic Juvenile Idiopathic Arthritis; NLR: Neutrophil-to-lymphocyte ratio
