# Supplementary material for: Development of a predictive tool for hemophagocytic lymphohistiocytosis/macrophage activation syndrome risk in adult-onset Still’s disease
Source: Front Immunol. 2026 Jul 31;17:1709136. doi: 10.3389/fimmu.2026.1709136 (PMC13473414; doi:10.3389/fimmu.2026.1709136)
Supplement: Supplementary file 4 [file Table3.docx]

Supplementary table 3. Missing data rates for candidate predictor variables before imputation (N = 190)

| **Variable** | **Missing, n** | **Missing %** |
| --- | --- | --- |
| Body temperature | 2 | 1.1 |
| White blood cell count | 0 | 0 |
| Neutrophil count | 0 | 0 |
| Lymphocyte count | 0 | 0 |
| Hemoglobin | 0 | 0 |
| Platelet count | 0 | 0 |
| C‑reactive protein | 2 | 1.1 |
| Fibrinogen | 0 | 0 |
| D‑dimer | 0 | 0 |
| APTT | 0 | 0 |
| PT | 0 | 0 |
| INR | 0 | 0 |
| ESR | 0 | 0 |
| Ferritin | 0 | 0 |
| sIL‑2R | 30 | 15.8 |
| ALT | 0 | 0 |
| AST | 0 | 0 |
| Bilirubin | 0 | 0 |
| Albumin | 0 | 0 |
| LDH | 0 | 0 |
| Systemic score | 0 | 0 |
| Response to high‑dose glucocorticoid | 0 | 0 |

Abbreviations: sIL-2R: soluble interleukin-2 receptor
