# Supplementary material for: Development of a predictive tool for hemophagocytic lymphohistiocytosis/macrophage activation syndrome risk in adult-onset Still’s disease
Source: Front Immunol. 2026 Jul 31;17:1709136. doi: 10.3389/fimmu.2026.1709136 (PMC13473414; doi:10.3389/fimmu.2026.1709136)
Supplement: Supplementary file 5 [file Table4.docx]

Supplementary table 4. Sensitivity analysis: complete-case vs. multiple imputation model for HLH/MAS risk

| **Predictor** | **Imputed model OR (95% CI)** | **Complete‑case OR (95% CI)** |
| --- | --- | --- |
| Body temperature | 1.43 (1.05–1.95) | 1.27 (0.92–1.77) |
| Platelet count | 0.994 (0.991–0.998) | 0.994 (0.991–0.998) |
| D‑dimer | 1.05 (0.99–1.11) | 1.045 (0.99–1.11) |
| Systemic score | 1.32 (1.04–1.68) | 1.421 (1.11–1.816) |
| Glucocorticoid response | 5.10 (2.23–11.70) | 5.468 (2.368–12.624) |
